# Valorization of Waste from Argan Seeds for Polyhydroxybutyrate Production Using Bacterial Strains Isolated from Argan Soils

**DOI:** 10.3390/polym15081972

**Published:** 2023-04-21

**Authors:** Amina Aragosa, Valeria Specchia, Mariaenrica Frigione

**Affiliations:** 1 Department of Biological and Environmental Sciences and Technologies, University of Salento, 73100 Lecce, Italy; amina.aragosa@unisalento.it (A.A.); valeria.specchia@unisalento.it (V.S.); 2 School of Science and Engineering, Al Akhawayn University, Ifrane 53000, Morocco; 3 Department of Innovation Engineering, University of Salento, 73100 Lecce, Italy

**Keywords:** polyhydroxybutyrate, bio-based polymers, argan soils, argan seed residue, PHB-producing bacteria

## Abstract

The aim of this work was to study the valorization of argan seed pulp, a waste material obtained from argan oil extraction, for the biosynthesis of polyhydroxybutyrate (PHB). A new species that showed the metabolic capacity for the conversion of argan waste into the bio-based polymer was isolated from an argan crop located in Teroudant, a southwestern region of Morocco, where the arid soil is exploited for goat grazing. The PHB accumulation efficiency of this new species was compared to the previously identified species 1B belonging to the genus Sphingomonas, and results were reported as dry cell weight residual biomass and PHB final yield measured. Temperature, incubation time, pH, NaCl concentration, nitrogen sources, residue concentrations, and culture medium volumes were analyzed with the aim of obtaining a maximum accumulation of PHB. UV-visible spectrophotometry and FTIR analysis confirmed that PHB was present in the material extracted from the bacterial culture. The results of this wide investigation indicated that the new isolated species 2D1 had a higher efficiency in PHB production compared to the previously identified strain 1B, which was isolated from a contaminated argan soil in Teroudant. PHB final yield of the two bacterial species, i.e., the new isolated and 1B, cultivated under optimal culture conditions, in 500 mL MSM enriched with 3% argan waste, were 21.40% (5.91 ± 0.16 g/L) and 8.16% (1.92 ± 0.23 g/L), respectively. For the new isolated strain, the result of the UV-visible spectrum indicates the absorbance at 248 nm, while the FTIR spectrum showed peaks at 1726 cm^−1^ and 1270 cm^−1^: these characteristic peaks indicated the presence of PHB in the extract. The data from the species 1B UV-visible and FTIR spectra were previously reported and were used in this study for a correlation analysis. Furthermore, additional peaks, uncharacteristic of standard PHB, suggest the presence of impurities (e.g., cell debris, solvent residues, biomass residues) that persisted after extraction. Therefore, a further enhancement of the sample purification during extraction is recommended for more accuracy in the chemical characterization. If 470,000 tons of argan fruit waste can be produced annually, and 3% of waste is consumed in 500 mL culture by 2D1 to produce 5.91 g/L (21.40%) of the bio-based polymer PHB, it can be estimated that the amount of putative PHB that can be extracted annually from the total argan fruit waste is about 2300 tons.

## 1. Introduction

The global consumption of plastic increased by 3.9% from 2012 to 2013 [1], and in 2016, the world produced 242 million tons of plastic waste, representing 12% of all solid municipal waste [2]. This high plastic production is followed by a corresponding high rate of plastic accumulation around the world. In 2015, the volume of global plastic waste reached 6.3 billion metric tons, and it is expected to grow to 12 billion tons by 2050 [3]. Conventional plastics, such as polyethylene (PE) and polypropylene (PP), take 20 to 100 years to be decomposed in nature, causing soil infertility, release of greenhouse gases and carcinogenic agents, and water and air pollution due to incineration and landfill disposal [4]. Because of their non-degradable nature, there is a growing awareness regarding the harmful consequences of plastic toxicity on the environment and its living things. Hence, there is an urgent need to develop alternative materials that can substitute petroleum-based plastics. This issue has raised interest in developing non-conventional plastics, such as bio-based polymers by using eco-friendly resources [1].

A bio-based polymer is a non-synthetic plastic that is biodegradable in nature, partially or totally produced from biological resources. Bio-based polymers are naturally occurring polyesters synthesized by a variety of microorganisms in abundant carbon sources and under limited conditions, e.g., lack of phosphorous, nitrogen, sulfur, and oxygen. Bio-based biodegradable polymers, in addition, can be easily deteriorated by microbial enzymes into CO_2_, H_2_O, and biomass under aerobic or anaerobic conditions, avoiding environmental contaminations [1].

Because of the good combination of mechanical, thermal, and chemical properties, bio-based polymers have a wide range of applications. Polyhydroxybutyrate (PHB), a microbial-derived polymer, lipid-like and water-insoluble molecule, member of the polyhydroxyalkanoate family (PHA), is biocompatible, 100% biodegradable, and hydrophobic. PHB is used for disposable packages, agricultural systems, medicines and medical devices, and drug delivery [5]. PHB is a thermoplastic semi-crystalline polymer, with a crystallinity degree ranging from 50 to 70%, displaying propylene-like physical and chemical properties, which makes it a promising bio-based polymer able to contribute to solve the environmental issues caused by synthetic not-biodegradable plastics [1]. Bio-based plastics production, including the PHB, accounts for 1% of the annual global plastic production. In 2019, the production capacity recorded by European Bioplastics and the Research Institute Nova-Institute was 2.11 million tons, and it is expected to increase to 2.43 million by 2024 [2].

Although the promising properties and advantages of the bio-based polymers, major drawbacks have been limiting their production and market. First of all, the pure microbial synthesized PHB showed some disadvantages, such as high fragility, low thermal stability, brittleness, and low nucleation density that limited its potential use for many years. Nevertheless, the development of copolymers, blended polymers, and chemical grafting increased the number of applications of the microbial-derived polymer in the last decades [4]. A second aspect to be considered for the large-scale production of PHB is the microbial strain. The best PHB producers identified among Gram-positive and -negative bacteria are *Bacillus* spp., *Ralstonia* spp., *Cupriavidus necator*, *Sphingomonas* spp., *Enterobacter aerogenes*, *Nocardia* spp., *Escherichia coli mutants*, *Azotobacter* spp., *Pseudomonas* spp., and *Alcaligenes* spp. However, their efficiency in the PHB synthesis depends upon the nature of carbon sources fermented along with other nutrients [6,7,8,9]. It has been demonstrated that, for a more efficient microbial synthesis of PHB, innovative research approaches should be implemented to reduce the costs of PHB extraction and purification, as well as to reduce the amount of secondary bio-products that affect the final yield of the bio-based polymer. Hence, more attempts should be made to employ genetic engineering microbes for more efficient carbon utilization [1]. Finally, the difficult replacement of petroleum-derived plastics with bio-based polymers is mainly due to a significant difference in market cost. The cost of PE or PP is 0.23 to 0.48 dollars per kilogram, while the cost of bio-based polymers is approximately 6 to 15 dollars per kilogram [1]. The high cost of bio-based polymers is primarily due to the expensive prize of raw material. In total, 70 to 80% of the cost of raw materials is represented by carbon sources used as feedstock for the microbial fermentation. Researchers are extending the experimentation to a sustainable use of agricultural waste residues as sole carbon sources for the bio-based polymer synthesis in order to reduce the production cost [1]. This experimental approach was demonstrated to be sustainable and reflects the principles of the circular economy: the raw material is inexpensive, renewable, available, and biodegradable [4]. Recent works investigated the efficient production of PHB from different agricultural materials, such as sugar cane waste [10], beet molasses [11], starch-based materials [12], corn steep liquor [13], crude palm kernel oil [14], waste glycerol [15], sunflower cake, soy and olive mill [16], malt wastes [17], tamarind kernel powder [18], and groundnut oil cake [19]. It is with this sustainable objective in mind that this work wanted to value the waste cake obtained from argan seed oil extraction as a costless, carbon-rich residue for PHB bacterial synthesis and extraction.

The argan tree (*Argania spinosa*) is an endemic species of the southwestern region of Morocco that covers an area of 3200 square miles called the arganraie, where 21 million trees are cultivated. These forests compose a unique landscape classified as biosphere reserves by UNESCO in 1998. The tree forest plays an important role of social, ecological, and economic value to the local population. In particular, the cosmetic and edible oil extracted from the argan seeds represents a valuable socio-economic resource for the country, which continuously aims to develop strategies for the conservation and domestication of the species [20]. The argan tree can reach 250 years of age, and it is able to resist the dry and arid soils of the region, ranging between 5° and 50 °C. The tree has multipurpose aspects—each part of the tree is usable and can be used as a source of income or food for the population: wood for fire and agricultural utensils, leaves as feedstock for grazing animals, and fruits for oil extraction. The fruit is 2 to 5 cm long, with a thick peel surrounding a sweet-smelling layer of pulpy pericarp. Inside, there is a very hard nut, which contains one to three oil-rich kernels with oval shape and brown color, used for cosmetic and edible oil production [21]. The residue of the fruit is represented by the external fruit pulp (pericarp), the nut, and the press cake waste obtained for oil extraction [22].

The aim of this study was, therefore, to present the promising capabilities of a new bacterial species isolated from argan soil to synthesize polyhydroxybutyrate by exploiting argan seeds waste, otherwise destined to disposal. Moreover, this work aimed to analyze how the adoption of optimal culture parameters enhances the final yield of PHB extracted, as well as how its preliminary chemical characteristics, determined by FTIR analysis, open the door to further examinations and potential applications of the microbial-based polymer. Finally, this research aimed to communicate how the characteristic arid and dry argan soils of the southwestern region of Morocco represent good reservoirs for any other PHB-producing bacteria that inhabit this natural source.

## 2. Materials and Methods

### 2.1. Dry Biomass and PHB Quantification

To carry out the production and extraction of putative polyhydroxybutyrate, the bacterial strain 2D, isolated from argan soil exploited for animal grazing [7], was cultivated in sterile Mineral Salt Medium (MSM, Merck, Darmstadt, Germany), a rich-glucose medium deprived of nitrogen sources, containing K_2_HPO_4_ (5 g/L), NaSO_4_ (0.5 g/L) MgSO_4_ · 7H_2_O (0.4 g/L), glucose (20 g/L), and 0.1% of mineral solution of the following salts: FeSO_4_ · 7H_2_O (2.8 g/L), MnCl_2_ · 4H_2_O (2 g/L), CoSO_4_ · 7H_2_0 (1.5 g/L), CuCl_2_ · 2H_2_0 (0.2 g/L), and ZnSO_4_ · 7H_2_0 (0.3 g/L) [23]. Initially, a subculture of the 2D1 strain was developed in 5 mL Luria–Bertani broth [24]. After 24 h of incubation at 37 °C, 1 mL of the 2D1 subculture was transferred in 50 mL Mineral Salt Medium at pH 7 in a 250 mL conical flask and incubated at 37 °C for 48 h [25].

#### 2.1.1. Dry Biomass Quantification

To determine the dry cell weight (DCW), the gravimetric analysis was implemented. After incubation time, the 50 mL culture of the 2D1 strain was centrifuged at 5000 rpm for 20 min (Hettich EBA 30 centrifuge, Labexchange, GmbH, Burladingen, Germany), and the pellet was washed twice with distilled water and finally dried at 100 °C to a constant weight [26]. The biomass quantification was calculated as reported in Equation (1). The residual biomass representing the difference of the dry cell weight (DCW) and the putative PHB extraction estimates the efficiency in the bio-based polymer production by the strain 2D1 grew in a glucose enriched medium [27].

Residual biomass (g/L) = DCW (g/L) − extracted putative PHB (g/L)(1)

#### 2.1.2. PHB Extraction and Purification by Sodium Hypochlorite—Chloroform Dispersion Method

For the putative PHB extraction, the Law and Slepecky method was implemented. The bio-based polymer was extracted from the same cell pellet previously obtained and used for the DCW quantification [28]. According to the dispersion method protocol, once the cell pellet was extracted from the 50 mL culture, it followed the digestion at 37 °C for 2 h with 30% sodium hypochlorite (Sigma-Aldrich, affiliated of Merck, KGaA, Damstadt, Germany). The mixture was then centrifuged at 5000 rpm for 20 min; the supernatant was discarded, and the pellet was sequentially washed with distilled water, acetone, and ethanol (Sigma-Aldrich, affiliated of Merck, KGaA, Damstadt, Germany). Finally, the pellet was dissolved in 5 mL hot chloroform (Sigma-Aldrich, affiliated of Merck, KGaA, Damstadt, Germany) and left overnight for the complete solvent evaporation at room temperature, and then the weight of the residual bio-based polymer was measured.

#### 2.1.3. PHB Quantification

The putative PHB accumulation by the strain 2D1 grew in MSM culture with glucose was weighed and recorded. The amount of putative PHB extracted from the strain 2D1 pellet was calculated [27], according to the following Equation (2):

extracted putative PHB (%) = extracted PHB (g/L)/DCW (g/L) × 100(2)

The estimations of the DCW, residual biomass, and bio-based polymer accumulation were also quantified in the presence of fructose, maltose, saccharose, sorbitol, lactose, xylose, and mannose incorporated one at a time in the MSM to replace glucose (Fisher Scientific, Goteborg, Sweden) [29].

Additional measurements of DCW, residual biomass, and PHB production were repeated by using a pretreated argan pulp, i.e., a residue obtained from the extraction of oil from the argan seeds [25].

### 2.2. Preparation and Chemical Pretreatment of Argan Seed Pulp

Once the argan kernels were pressed for the oil extraction, the remaining residue was collected and pretreated for the bacterial growth and PHB synthesis [30]. In this work, the argan kernel waste used for the fermentation process was the roasted residue obtained from the edible argan oil extraction, which was donated by the “Cooperative Feminine Amagour Argan”, one of the argan cooperative factories located in the southwestern region of Morocco. The argan residue was dried at 65 °C for 24 h in the oven (Heratherm™ General Protocol Ovens—230VAC 50/60 Hz, Carthage, MO, USA) and afterwards ground it at 4500 rpm for 5 min in a blender (Waring laboratory blender, Sigma-Aldrich, St. Louis, MO, USA). Among all the particles size, only the 0.4 mm ground particles were selected to prepare the optimal nutrients enrichment. A total of 20 g of the 0.4 mm particles was treated with NaOH and Ca(OH)_2_ solutions (0.5, 1, 2% *w*/*v*) (Merck, Darmstadt, Germany), autoclaved for 15 min at 121 °C (Systec V-40, Systec GmbH, Linden, Germany), and filtered with Whatman filter paper (N°1, pore size 0.11 µm, Sigma-Aldrich, St. Louis, MO, USA). The residue sample was then washed with sterile water, dried in the oven at 80 °C for 24 h, and neutralized with H_2_SO_4_ and H_3_PO_4_ solutions (1,2,3%, *v*/*v*) (Merck, Darmstadt, Germany). Once filtered again, the sample’s final pH was adjusted to 7 by adding NaOH [31]. The pretreated residue sample obtained was then used in MSM culture, at a concentration of 20 g/L for the 2D1 strain growth and PHB synthesis. DCW, residual biomass, and PHB measurements were appreciated and are reported in the Section 3.

### 2.3. Cultivation Conditions for Maximum Bacterial Growth and PHB Production

In this work, the efficacy of the isolated 2D1 strain was evaluated for PHB production. To maximize the bacterial cell growth and the PHB synthesis, the following cultivation parameters were studied one at a time: temperature and incubation time, pH, NaCl concentration, nitrogen sources, biomass concentration, and culture volume [32].

#### 2.3.1. Effect of Temperature and Incubation Time

The selected isolate 2D1 was subcultured in 5 mL Luria–Bertani broth and incubated at 37 °C for 24 h. A total of 1 mL of the subculture was transferred in 250 mL conical flask with 50 mL MSM containing 1% pretreated argan seed residue. The effect of temperature and incubation time on the DCW and PHB production was determined by conducting sequential bacterial growths at 20°, 24°, 28°, 32°, 36°, and 40 °C for 72 h of incubation for each growth. Spectrophotometric analysis (Jenway6320D, Fisher Scientific, Leichestershire, UK) at 600 nm was determined for each selected temperature every 12 h of incubation. The maximum optical density measured was used to select the optimal temperature and incubation time of the strain, while the DCW and the putative PHB production were measured, and the PHB % was calculated as per the above Equation (2) [33,34].

#### 2.3.2. Effect of pH

For the selection of the best pH medium, 1 mL of the 2D1 subculture in Luria–Bertani broth was transferred and grown in 250 mL conical flasks containing 50 mL MSM enriched with 1% pretreated argan seed residue at different pH values: 4.5, 5.5, 6.5, 7.5, 8.5, 9.5, and 10.5. The culture was incubated at the temperature and incubation time selected earlier. The DCW and PHB extracted at each pH value were measured, and the PHB % was calculated according to Equation (2) [25].

#### 2.3.3. Effect of NaCl Concentration

For the selection of the best NaCl concentration in the medium, 1 mL of the 2D1 subculture in Luria–Bertani broth was transferred and grown in 250 mL conical flasks containing 50 mL MSM enriched with 1% pretreated argan seed residue at different NaCl concentrations, from 0 to 20%. The culture was grown at the temperature, incubation time, and pH selected earlier. The DCW and PHB extracted at each NaCl concentration were measured, and PHB % was calculated as per the above Equation (2) [25].

#### 2.3.4. Effect of Nitrogen Sources

For the selection of the best nitrogen sources, 1 mL of the 2D1 subculture in Luria–Bertani broth was transferred and grown in 250 mL conical flasks containing 50 mL MSM enriched with 1% pretreated argan seed residue containing 0.25% of different nitrogen sources: NH_4_Cl, NH_4_NO_3_, (NH_4_)_2_SO_4_, peptone, tryptone, beef extract, and yeast extract. The culture was grown at the temperature, incubation time, pH, and NaCl concentration selected earlier. The DCW and PHB extracted from different nitrogen sources were measured, and PHB % was calculated as per the above Equation (2) [25].

#### 2.3.5. Effect of Argan Seed Waste Concentration

Moreover, different argan seed residue concentrations were assessed. Once again by starting from a subculture of the 2D1 strain in Luria–Bertani broth incubated for 24 h at 37 °C, 1 mL of the subculture was transferred in 250 mL flasks with 50 mL MSM. Each flask was supplemented with increasing concentrations of the pretreated argan seed waste: 0.5, 1, 2, 3, 4% (*w/w*). The culture was grown with all previously selected optimal conditions implemented for temperature, incubation time, pH, NaCl concentration, and nitrogen sources [35]. The DCW and PHB extracted at different argan seed waste concentrations were measured. The PHB % was calculated as per the above Equation (2).

#### 2.3.6. Effect of Culture Fermentation Volume

Finally, a larger culture fermentation volume was assessed. A total of 5 mL of the 2D1 strain subculture at 37 °C for 24 h was transferred in a 1 L conical flask containing 500 mL MSM enriched by pretreated argan residue. The larger culture volume was grown with all previously selected optimal parameters of temperature, incubation time, pH, NaCl concentration, nitrogen sources, and argan residue concentration [35]. All optimal growth conditions selected earlier were applied in the larger culture fermentation volume. The DCW and PHB extracted at different argan waste concentrations were measured. The PHB % was calculated as per the above Equation (2).

### 2.4. Characterization of Extracted PHB through Analytical Tools

The extracted putative PHB from 500 mL 2D1 culture in MSM enriched with 3% argan seed waste was characterized as follows, and the data were obtained compared to standard PHB.

#### 2.4.1. Preliminary Characterization by UV-Visible Spectrophotometry

A preliminary identification of the extracted putative PHB was conducted by UV-visible spectrophotometry. Initially, the extract was digested with H_2_SO_4_ at 100 °C for 10 min [36], to be afterwards analyzed at 235 nm in a range of 200 to 800 nm using the UV-visible spectrophotometer (V-530, Jasco, Tokyo, Japan) using H_2_SO_4_ as blank [8,37].

#### 2.4.2. Chemical Characterization by Fourier Transform Infrared Spectroscopy (FTIR)

The functional groups CH, CH_2_, CH_3_, C=O, and C-O, which are significant for the presence of PHB in the extracted material, were identified and detected using FTIR analysis. The extracted material, subjected to a lyophilization process in order to completely evaporate the chloroform, was analyzed by a FTIR spectroscopy (JASCO FTIR-6300, Tokyo, Japan) over a range of 4000 to 400 wavenumber/cm, placing the samples on K-Br discs [36]. The detected functional groups were analyzed to confirm that the extracted bio-based polymer was the PHB [37].

### 2.5. Statistical Analysis

All experiments were conducted in triplicate. The results reported as mean and ± standard deviation were subjected to basic statistical analysis of variance (one-way ANOVA) using the commercial statistical software OriginPro 9.0. A significant difference was considered for *p* < 0.05 with a 95% confidence interval [6].

## 3. Results and Discussion

### 3.1. Dry Biomass and PHB Quantification

**Figure 1:**
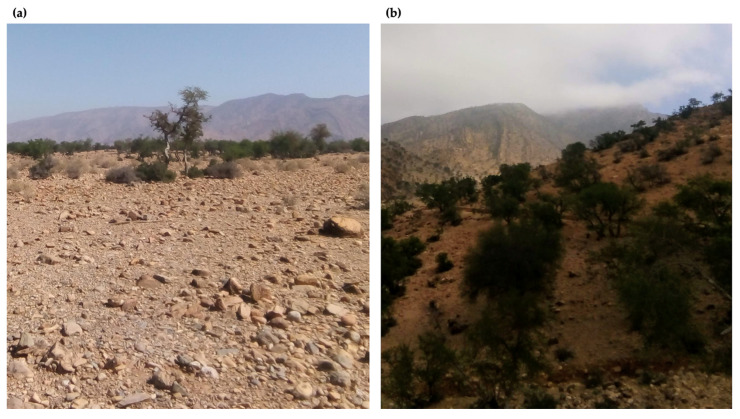
(**a**) Geomorphology in the southwestern region of Morocco, where the argan soil is dry and arid; (**b**) argan tree crop in the valley of Teroudant.

The bacterial species 2D1 was previously isolated from the argan soil of a crop used for extensive grazing exploitation and identified as a PHB producer [7]. The crop for the bacterial isolation was located in a valley of the Teroudant region, where the soil is extremely dry and arid, and the deep-rooted argan trees prevent soil erosion and maintain water resources [20]. It is not the first time that PHB-producing bacteria have been isolated from unusual soils with difficult geomorphology and climate conditions, contaminated, and exploited. Good few examples include soils contaminated by sewage [38]; industrial effluent discharge sites [39]; cattle rumen fluid [40]; cow dung [41]; and dumping, industrial, and animal waste sites [42], as well as unusual soil sources such as lake soil [43], nursery field (Biradar et al., 2015) [33], hypersaline lake soil [44], and Antarctic environments [45]. Figure 1 shows the geomorphological features of the site for the soil sample collection of this work (Figure 1a), and an example of the argan trees crop in Teroudant region (Figure 1b).

The isolated bacterial species 2D1 was already identified as a putative PHB producer by staining analysis in the previous work [7]. In this research, DCW and PHB quantification were appreciated in the 50 mL culture enriched with 1 g of glucose as a carbon source, as well as with supplementary sugars (fructose, maltose, saccharose, sorbitol, lactose, mannose, and xylose) used one at a time to replace glucose in the medium. All measurements were repeated in triplet for each sugar, and the mean, standard deviation (SD), and variance were measured. As reported in Table 1, the highest PHB yield measured for the species 2D1 was in the enrichment with glucose, saccharose, and xylose, with calculated values of bio-based polymer extracted of 0.95 ± 0.12 g/L (5.5%), 0.88 ± 0.13 g/L (4.14%), and 0.83 ± 0.08 g/L (4.14%), respectively.

**Figure 2:**
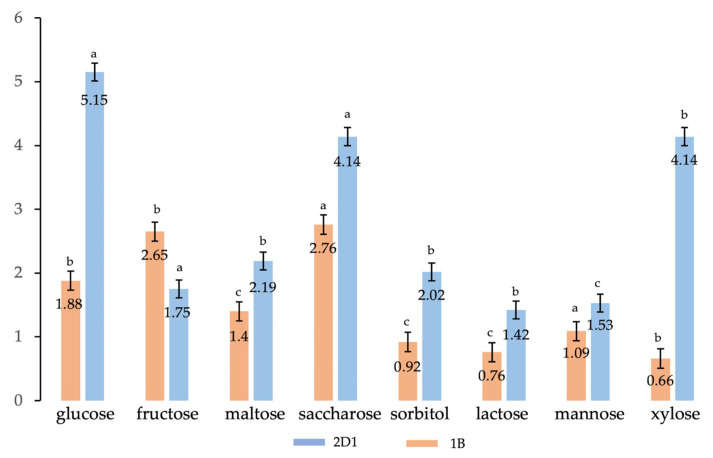
Effect of different carbon sources on PHB % accumulation by the species 2D1 (in blue) and 1B (in orange); results are calculated as mean of triplicates ± standard error; a,b,c letters indicate significant differences at the *p* < 0.05 (one-way ANOVA) using OriginPro9.0.

These obtained results were compared to the PHB final yield measured for the first isolated and identified species from the argan tree crop. This first species named 1B was isolated from an argan tree crop located in Teroudant valley, in the proximity of an urban area contaminated by wastewater and garbage [7]. The species 1B was identified as a new PHB-producing strain belonging to the genus *Sphingomonas* for the first time isolated from argan crop soil, and the DCW, residual biomass, and putative PHB production were measured [8]. The correlation between the two strains, the new isolated 2D1, and the previously identified 1B, reported in Figure 2, indicates how the new isolated species (in blue) has a higher PHB final yield compared to the species 1B (in orange) in all sugar enrichments tested at standard growth conditions of 37 °C for 24 h of incubation. Moreover, it can be appreciated how both species led to a high putative PHB final yield in the presence of saccharose, 4.14% (0.88 ± 0.13 g/L) for 2D1 and 2.76% (0.58 ± 0.22 g/L) for 1B, while unlike 1B, the new species 2D1 showed better productions of putative PHB with glucose and xylose corresponding to 5.15% (0.95 ± 0.12 g/L) and 4.14% (0.83 ± 0.08 g/L), respectively, against the 1.88% (0.57 ± 0.06 g/L) and 0.66% (0.10 ± 0.22 g/L) produced by 1B.

However, implementing the fermentation process of the promising strain 2D1 from a 50 mL culture to a large-scale process involves excessive sugar consumption with high costs of production. The utilization of inexpensive carbon sources has become one of the significant research projects in PHB production [5]. A wide variety of agro-industrial residues represent attractive candidates because of their low cost, availability, favorable chemical composition, and high PHB production [46]. The promising PHB production obtained by the strain 2D1 with sugars pushed this work to investigate the species ability to grow and produce PHB in a fermentation medium enriched with pretreated argan seed waste. Three reasons mainly focused this work on the use of argan seed residue as agricultural waste. First, both microorganisms, 2D1 and 1B, were isolated from argan soils, a natural reservoir where the strains have adapted to ferment the argan fruit residue. Second, the argan seed waste represents a carbon-rich and inexpensive residue that can be used for bacterial fermentation, reducing the PHB production and the waste disposal costs. Third, the already isolated species 1B identified as belonging to the genus *Sphingomonas* suggests that the new isolated strain 2D1 might be an endemic species that grows in the argan tree soil, in a particularly unique environment characterized by dry and hot conditions. Argan seed waste is a residue obtained from argan oil extraction, which besides its fatty acids is rich in carbohydrates, such as glucose, fructose, saccharose, arabinose, xylose, and rhamnose [47]. The waste donated by “Coopérative feminine Amagour Argan” was pretreated by draying, grounding, and filtering the raw material for better solubility. A total of 1 mL of a 2D1 subculture in Luria–Bertani broth was inoculated in a 50 mL MSM enriched with 1% (20 g/L) of pretreated argan seeds waste. After the culture was incubated at 37 °C for 48 h, the DCW, residual biomass, and PHB extracted were measured according to Equations (1) and (2), as reported in the Section 2. Table 2 reports the measurements obtained from the new isolated strain 2D1 compared with the already published results for the strain 1B.

Although both species led to a very similar amount of dry cell weight in the same culture conditions, the quantity of bio-based polymer extracted from 2D1 tripled the quantity extracted from 1B. This bacterial behavior led to the notion that the new isolated strain 2D1 achieved a higher synthesis capability or showed a greater efficiency in the use of the waste. Comparable results of high efficiency production of PHB were already reported in literature for the species *Cupriavidus necator* [31]. Through the coupling of citric molasses fermentation and pretreated extraction methods using propylene carbonate, *Cupriavidus necator* productivity increased five times, while the production costs were reduced by 18% (Pavan et al., 2019) [48]. Aramvash et al. also reported the highest accumulation of PHB by *Cupriavidus necator* in optimal conditions using fructose as a source of carbon corresponding to 7.48 g/L [49]. The attractive aspects of the new species 2D1 induced this study to investigate the optimal growth parameters of the strain for an increment in the PHB extraction [5,6,42].

### 3.2. Cultivation Conditions for Maximum Bacterial Growth and PHB Production

#### 3.2.1. Effect of Temperature and Incubation Time

**Figure 3:**
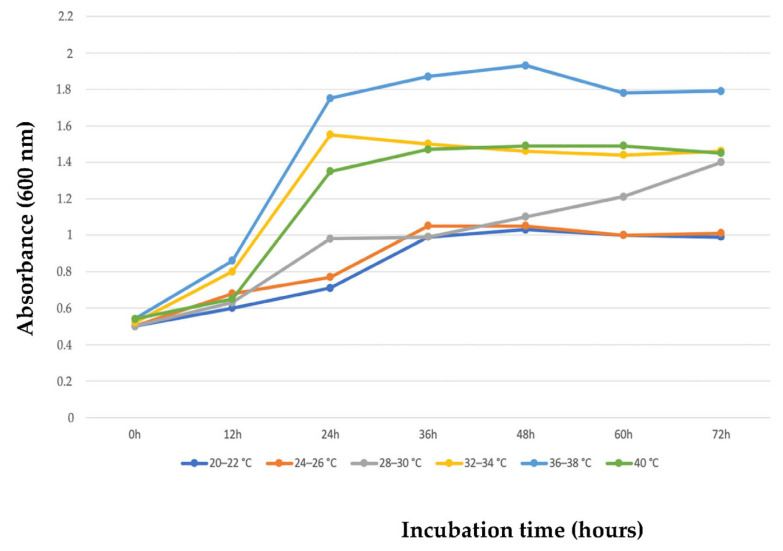
Optical density of the strain 2D1 measured at different temperatures over an incubation time interval of 72 h. The strain showed a better growth at 36° to 38 °C in 48 h of incubation with an OD of 1.93.

The cultivation conditions investigated for efficient PHB accumulation by the species 2D1 were the temperature and the incubation time, the different NaCl concentrations, the pH, the different nitrogen sources, the different concentrations of argan seed waste, and the volume of culture medium [25]. The incubation temperature and time represent major factors for the bacterial growth and PHB synthesis. In particular, the temperature affects the regulatory enzymes (beta-ketothiolase, acetoacetyl-CoA reductase, and PHB synthase) and their activity for the PHB synthesis [50]. The 2D1 strain was cultivated in 50 mL MSM medium enriched with 1% pretreated argan seed residue and incubated at different temperatures, from 20° to 40 °C, and the optical density (OD) was measured every 12 h for 72 h in order to determine the favorable range of temperature and incubation time of 2D1. As presented in Figure 3, it can be appreciated how the strain 2D1 had the highest OD (1.93) between 36° and 38 °C after 48 h of incubation. These selected parameters represent the best temperature and incubation time at which the species 2D1 had the highest putative PHB accumulation. At these optimal conditions, the DCW measurement and putative PHB yield were 21.16 ± 0.25 g/L and 9.03% (1.91 ± 0.23 g/L), respectively.

**Figure 4:**
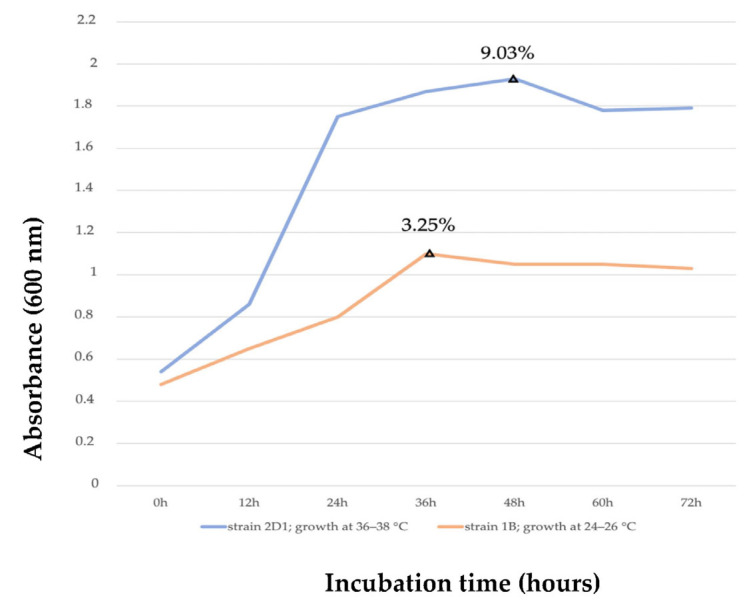
Growth curve correlation of the new isolated species 2D1 (in blue) with the previously identified species 1B (in orange) and their respective putative PHB yield at their maximal growth: 2D1 reported an average putative PHB yield of 9.03% at 48 h of incubation; 1B reported an average putative PHB yield of 3.25% at 36 h of incubation.

Besides the determination of the optimal temperature and incubation time for the species 2D1, the DCW and PHB measured confirmed the species capacity on the putative PHB accumulation by using argan seed waste, as earlier reported in Table 2. The efficient growth of the strain 2D1 can be more valued when compared with the bacterial growth of strain 1B as reported in Figure 4. A straightforward comparison of the two species (2D1 in blue, 1B in orange) indicated their own different incubation temperatures, as well as PHB accumulation efficiency over 72 h of incubation. 1B grew better between 24° and 26 °C for 30 h of incubation with an average PHB yield of 3.25% (0.66 ± 0.19 g/L), while 2D1 showed the most efficient growth between 36° and 38 °C for 48 h of incubation with an average PHB yield of 9.03% (1.91 ± 0.23 g/L). It can be noticed how 2D1 showed a higher OD, particularly from 12 to 60 h of incubation, wherein the 2D1 growth was around twice that of 1B. The greater optical density corresponded to a higher cell density in the culture, which can suggest a greater amount of putative PHB accumulated by the cells [32]. With this expectation in mind, it was necessary to explore the other variables: NaCl concentration, pH, nitrogen sources, argan residue concentration, and different culture volumes for a more efficient growth and productivity of 2D1 [51].

#### 3.2.2. Effect of pH

The optimal pH was determined in the range of 4.5 to 10.5, and the DCW and putative PHB yield were measured at intervals of 1. The optimal pH identified for the species was in the range of 7.5 to 8.5, wherein the maximum DCW and PHB accumulation measured corresponded to 22.67 ± 0.17 g/L and 2.04 ± 0.13 g/L, respectively, corresponding to 8.99% of the putative PHB. This is consistent with what is reported in the literature. A neutral pH value is generally suitable for PHB production, while an acidic pH has no effect on the polymer synthesis, and an alkaline value depolymerizes the polymer [32]. The pH value selected was maintained as an optimal condition in the culture media for the following selected parameters to be tested.

#### 3.2.3. Effect of NaCl Concentration

The optimal NaCl concentration was measured from 0 to 10% with measurements taken every 1% interval. The strain 2D1 showed the highest DCW and PHB production at 10% of NaCl in the culture media, with 23.75 ± 0.19 g/L and 2.86 ± 0.13 g/L, respectively, corresponding to 12% of the PHB final yield. Several species have been reported in the literature as able to grow at high salt concentrations, such as *Haloferax mediterranei* and *Haloferax volcanii* (Archeae domain), which are able to grow above 20% of NaCl [52]. The results obtained for 2D1 suggest that the species was adapted to a soil whose composition is particularly concentrated in salts. Indeed, according to Chakhchar et al., argan forests grow on shales, quartzite, limestone, and alluvium soils rich in minerals [21].

#### 3.2.4. Effect of Nitrogen Sources

Ammonium chloride, ammonium nitrate, ammonium sulphate, peptone, tryptone, beef extract, and yeast extract were the nitrogen sources tested for the selection of the best source of nitrogen [35]. A total of 0.25% of each nitrogen source was added to the 50 mL culture of MSM enriched with 1% pretreated argan waste, and DCW and PHB production were calculated after growth. The favorable nitrogen compounds selected for the highest bio-based polymer extracted were ammonium chloride and ammonium nitrate, with DCW and PHB accumulations of 23.45 ± 0.32 g/L and 2.64 ± 0.26 g/L, respectively, for ammonium sulfate, and 23.58 ± 0.30 g/L and 2.96 ± 0.25 g/L, respectively, for ammonium nitrate. The PHB final yield extracted was 11.25% with ammonium chloride and 12.55% with ammonium nitrate.

#### 3.2.5. Effect of Argan Seeds Waste Concentration

To maximize the cell productivity for a higher extraction of putative PHB, increasing concentrations of argan seed residue were implemented [35]. All selected parameters so far were determined in 50 mL MSM enriched with 1 g/L (1%) of pretreated argan waste. Other fermentations were conducted in the same medium volume but by adding increasing concentrations of the waste: 2 g/L, 3 g/L, 4 g/L, and 5 g/L. A maximum putative PHB extraction of 14.02% was measured at 3 g/L of argan residue, wherein the corresponding DCW and PHB measured were 23.25 ± 0.25 g/L and 3.26 ± 0.20 g/L, respectively. These results are in agreement with what was reported by Saleem et al., when the bacterial species produced 2.75 g/L of PHB by fermenting 3% of maltose as a carbon source [35].

#### 3.2.6. Effect of Culture Fermentation Volume

The last condition to be tested in this work to optimize the culture conditions that increment bacterial growth and bio-based polymer accumulation was the volume of the culture medium. Up until now, all extractions were conducted on 50 mL MSM culture with all previously selected nutritional and physicochemical conditions implemented. The literature indicates that optimal culture volumes for PHB synthesis range from a 100 mL [2,53,54] to 500 mL fermentation batch [55]. Hence, a larger culture volume was adopted using a 500 mL culture in a 1000 mL conical flask, again respecting all optimal growth parameters selected previously. Experiments in 500 mL culture were performed in triplet, and the mean, standard deviation, and variance measured for DCW and putative PHB extracted were, respectively, 27.61 ± 0.09 g/L and 5.91 ± 0.16 g/L for a putative PHB accumulation of 21.40%.

All the selected optimal growth conditions for the species 2D1, with the corresponding DCW, bio-based polymer accumulation, and final yield measured, are summarized in Table 3.

#### 3.2.7. Comparative Analysis of Optimal Growth Conditions and Putative PHB Accumulation of the Species 2D1 and 1B

**Figure 5:**
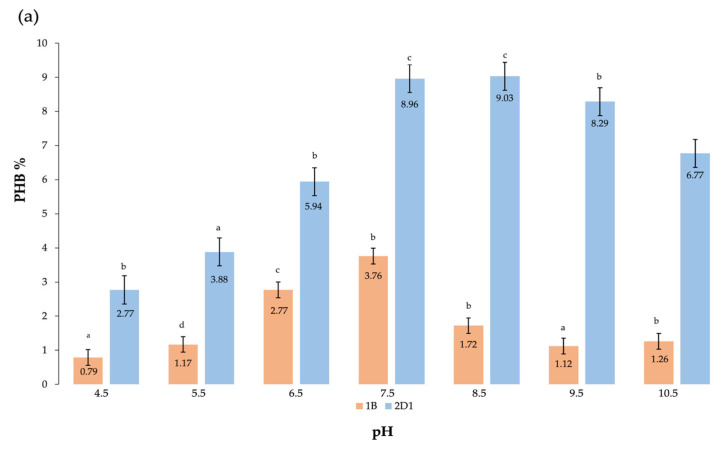

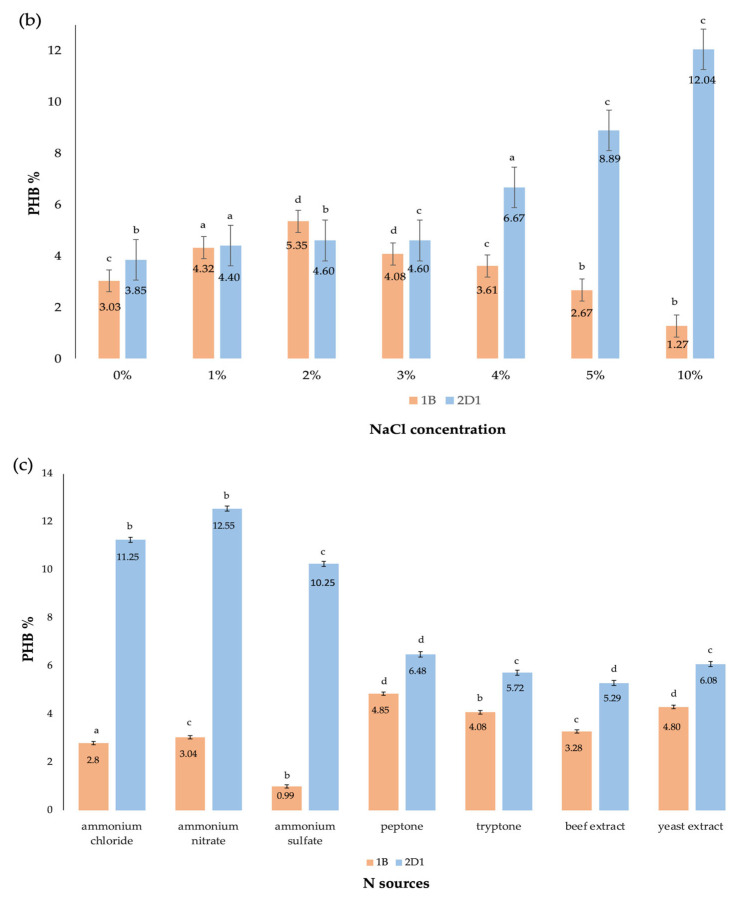
Correlation of the biobased polymer yield for the new isolated species 2D1 (in blue) and the previously identified species 1B (in orange) at different (**a**) pH values, (**b**) NaCl concentrations, and (**c**) N sources. The results are calculated as mean of triplicates ± standard error; a,b,c,d letters indicate significant differences at *p* < 0.05 (one-way ANOVA) using OriginPro9.0.

Metabolic processes, such as PHB synthesis, are very susceptible to slight physicochemical changes. To better appreciate the bio-based polymer accumulation efficiency of the new isolated species 2D1, a correspondence analysis of data was constructed to better understand the strain accumulation efficiency for a large-scale production. By comparing the new isolated species 2D1 with the already identified 1B, it is remarkable how 2D1 shows a higher efficiency to produce the bio-based polymer. In Figure 5a, the results show the putative PHB extraction from both species at different pH values, and besides the much greater amount of bio-based polymer produced by 2D1, it can be appreciated how the two species show similar optimal pH values for the synthesis of putative PHB. While 2D1 produced a maximum final yield of 9.03% of PHB at pH 8.5, the 1B produced only 3.76% of PHB at pH 7.5. On the other hand, when the two species were tested for the polymer synthesis at different NaCl concentrations, a different behavior was identified. The putative PHB extracted from 2D1 at an optimal NaCl concentration of 10% corresponded to 12.04%, while the extraction from 1B at the optimal concentration of 2% corresponded to 5.35%, which was about half of what was produced by the new isolated 2D1. Another chemical comparison was the bacterial growth and PHB synthesis in the presence of nitrogen sources. Among all the tested nitrogen sources, the yields of putative PHB produced were 10.25%, 11.25%, and 12.55% for ammonium sulfate, ammonium chloride, and ammonium nitrate, respectively. The yields of putative PHB for 1B were 4.80% and 4.85% for yeast extract and peptone, respectively. Once again, also for the selection of nitrogen sources, it was ascertained that PHB production of 2D1 was more than double that of 1B with the favorable nitrogen source added to the growth medium.

**Figure 6:**
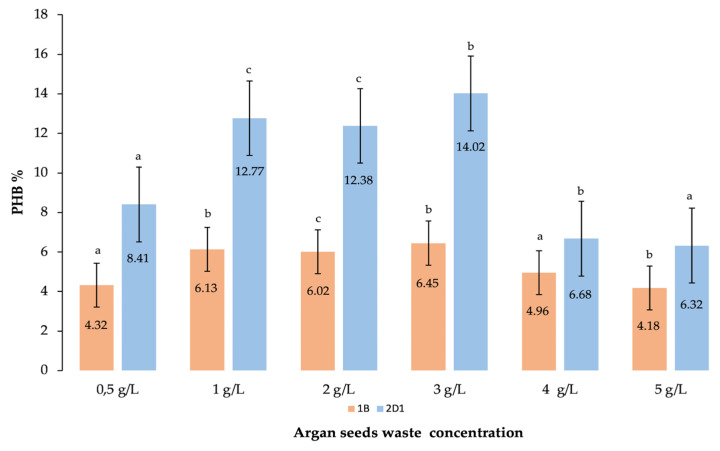
Correlation of the biobased polymer yield measured for the new isolated species 2D1 (in blue) and the previously identified species 1B (in orange) when different concentrations or argan seed waste were used to enrich the 50 mL MSM culture. The results are calculated as mean of triplicates ± standard error; a,b,c letters indicate significant differences at *p* < 0.05 (one-way ANOVA) using OriginPro9.0.

Afterwards, different residue concentrations were tested for maximum PHB production. According to Chekroud et al., in their study of PHB synthesis using date syrup, the PHB final yield measured was directly proportional to the date syrup concentration added to the medium. In particular, the PHB final yield increased from 26.06% at 2% of date syrup to 32.62% at 8% syrup because of the high sugar content in the concentrated residue [6]. Figure 6 shows a considerable rise in bio-based polymer formation by 2D1 with increasing concentrations of argan waste, while the species 1B showed a less noteworthy change.

For species 1B, the results indicated a slight increase in bio-based polymer extraction from 1 to 3% of residue, while a noticeable increment was indicated in the results for the species 2D1. However, for both strains, the results indicated a drastic drop in the putative PHB production when the concentration of waste increased to 4 and 5%. This can be explained by the inefficiency of the species to completely consume the carbon-rich residue, or because of the incomplete solubility of the residue in the medium. However, the production cost for the PHB extraction is particularly focused on the use of inexpensive raw biomass. Several studies are focused on sugar-rich agriculture waste as attractive candidates for bio-based polymer synthesis [5]. With this objective, this study wanted to estimate the amount of waste produced from the argan harvesting. The average fruit yield in Morocco corresponds to 30 kg/tree/year. One fruit’s average weight ranges from 5 to 20 g. The argan pulp represents 55 to 75% of the fruit fresh weight, while only the rest of the 25 to 45% is represented by the nut and seed [22]. The absorption qualities of materials made from argan nutshells have already been reported in the literature. Sawdust bioabsorbent material capable of removing heavy metals and uranium in fertilizer manufacture (Qamouche et al., 2021) or mesoporous material used for wastewater treatment (Zbair et al., 2018) are two examples [56,57]. Furthermore, hard carbon derived from argan nutshells can be used to create appealing negative electrodes for sodium-ion batteries (Dahbi et al., 2017) [58]. The seeds are pressed for the argan oil extraction, and the pressing cake resulting from the extraction represents the waste used for this study (Machqoq et al., 2021) [47]. The average of 65% of fruit pulp and 10% of press cake represents a total of 75% (an average of 9.4 g of fruit) of agricultural waste that can be obtained from each fruit. This value can be estimated as a total of 470,000 tons of argan waste produced annually by the total amount of trees cultivated in the southwestern region of Morocco (data provided by “Coopérative feminine Amagour Argan”) [59].

**Figure 7:**
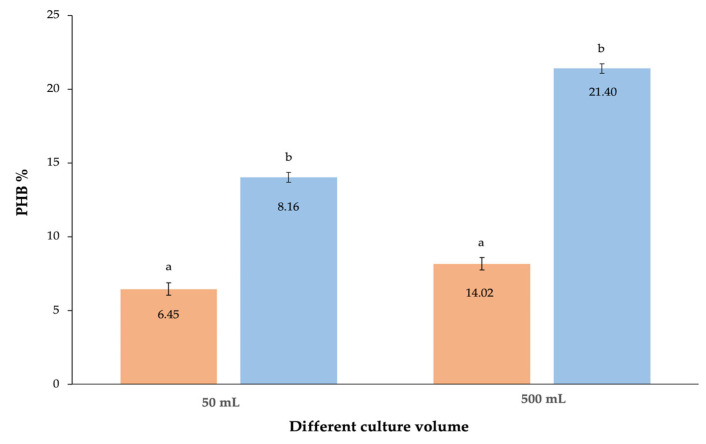
Correlation of the biobased polymer yield measured for the new isolated species 2D1 (in blue) and the previously identified species 1B (in orange) at different culture volumes: 50 mL and 500 mL. The results are calculated as mean of triplicates ± standard error; a,b letters indicate significant differences at the *p* < 0.05 (one-way ANOVA) using OriginPro9.0.

To complete the study on the growth optimization of the new species, 2D1, the fermentation capabilities were studied by implementing a larger culture volume. As shown in Figure 7a, a significant increase in PHB accumulation by 2D1 was observed from 50 mL (8.16%) to 500 mL (21.40%) culture volumes. This increase was appreciated also for the species 1B, wherein the PHB final yield increased from 6.45% at 50 mL culture to 14.02% for a 500 mL culture. However, the increase in 1B putative PHB yield was less remarkable than what was observed for 2D1. If 470,000 tons of argan fruit waste can be produced annually, and 3 g/L of waste is consumed in 500 mL culture by 2D1 to produce 5.91 g/L (21.40%) of bio-based polymer PHB, it can be estimated that the amount of putative PHB that is extracted annually from the total argan fruit waste is about 2300 tons.

## 4. Characterization of Extracted PHB through Analytical Tools

### 4.1. Preliminary Characterization by UV-Visible Spectrophotometry

**Figure 8:**
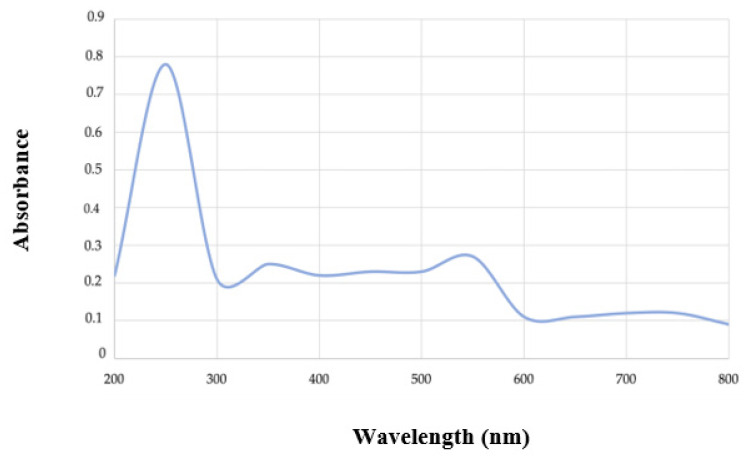
UV-visible spectrum of putative PHB extracted from strain 2D1. A characteristic peak of 0.78 absorbance at 248 nm confirms the presence of PHB in the extracted material.

The bio-based polymer extracted from 2D1 was used for preliminary identification in UV-visible spectrophotometry at a range of 200 to 800 nm. The spectrum obtained by this analysis is reported in Figure 8. A peak of 0.78 absorbance was observed at 248 nm. This peak is reported to be characteristic of the PHB polymer reported by Sayyed et al. (2010) and Giedraityte et al. (2015) [36,37]. This result strongly supports the presence of PHB in the extracted material.

### 4.2. Chemical Characterization by Fourier Transform Infrared Spectroscopy (FTIR)

**Figure 9:**
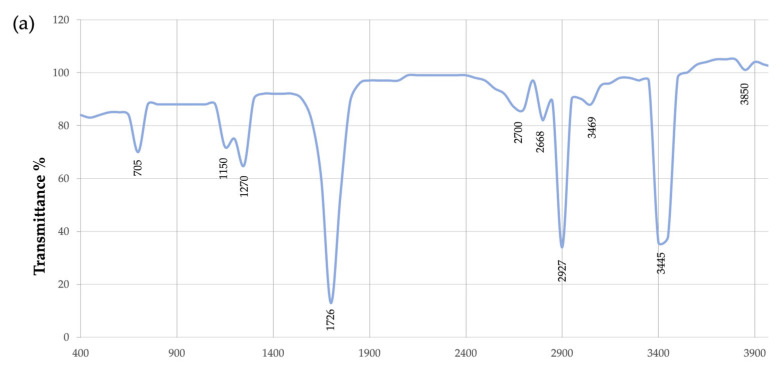

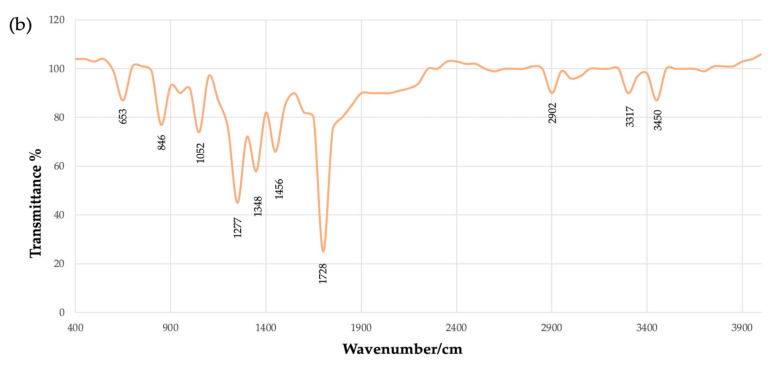
FTIR spectrum of putative PHB extracted from strains 2D1 (**a**) and 1B (**b**). 2D1 and 1B show characteristic peaks of PHB: at 1726 cm^−1^ (**a**) and 1728 cm^−1^ (**b**), respectively, corresponding to C=0 ester group; at 1270 cm^−1^ (**a**) and 1277 cm^−1^ (**b**), respectively, corresponding to CH groups.

The Fourier infrared spectroscopy technique is generally used to characterize the functional groups present in the extract. Because of the technique accuracy, reproducibility, small sample, and absence of solvents, FTIR analysis emerged as a reliable approach for chemical characterization (Lathwal et al. (2018)) [60]. The polymer extracted from 2D1 was analyzed by FTIR analysis in a frequency range of 400 to 4000 wave number cm^−1^ to identify the functional groups of the extract (Figure 9). For the 2D1 extract (Figure 9a), a wide range of the absorption peak was observed at 3445 cm^−1^, indicating the presence of -OH group bonds, followed by a second transmittance peak at 2927 cm^−1^, generally related to the symmetric C-H stretching of the CH_2_ groups of fatty acids [51]. The sharp peak at 1726 corresponded to the C=O ester group characteristic of PHB, while the short peak at 1270 cm^−1^ indicated the presence of CH groups [61,62]. Unlike the latter, all observed peaks can confirm that the extracted material from 2D1 culture contained the bio-based polymer PHB. This analysis confirms what the staining tests have already revealed: the presence of bacterial endospores and PHB intracellular granules was detected by methylene blue, malachite green, and Sudan black staining in the previous study [7]. Nevertheless, the peak at 705 cm^−1^, as well as other short peaks observed (e.g., 1150, 2650, and 3850) that have no correlation with the characteristic peaks of the standard PHB, can be associated to the presence of impurities in the extract. Moreover, the chemical analysis by FTIR of the previously identified species 1B presented characteristic peaks of PHB at 1728 cm^−1^ and 1277 cm^−1^ (Figure 9b), but again many other short peaks at 1052, 3317, and 3450 wavenumber/cm were incongruent with the standard PHB spectrum. Once again, this second FTIR analysis of a different extract sample, even though it confirms the presence of PHB in the extract, indicates that an enhancement of the extraction method has to be implemented in order to guarantee a more accurate analysis of the purified sample.

## 5. Conclusions

The promising strain 2D1, isolated from argan tree soil exploited for animal grazing, and previously identified as a PHB producer, was cultivated in MSM and sugar addition: glucose, fructose, maltose, saccharose, mannitol, sorbitol, lactose, and xylose. The species was able to ferment all sugars, particularly glucose, saccharose, and xylose, with putative PHB final yields of 5.14%, 4.14%, and 4.14%, respectively. The promising results obtained with simple sugars induced this work to investigate the ability of 2D1 to ferment a rich-sugar biomass, represented by argan seed pulp, a costless waste material obtained from argan oil extraction. The highest putative PHB final yield extracted from 2D1 culture corresponded to 9.19% in MSM enriched with 1% argan seed waste. The already identified species 1B as belonging to the genus *Sphingomonas*, whose PHB accumulation properties were studied previously, was able to accumulate 3.06% of the bio-based polymer in the same culture conditions. The work continued by investigating the optimal culture conditions of the species to be selected to maximize the putative PHB extraction and to value the use of the residue. Among all the parameters implemented, the 2D1 strain demonstrated optimal growth between 36° and 38 °C for a 48 h incubation, at pH 7, with a NaCl concentration of 10%, and in the presence of ammonium nitrate as a nitrogen source. The strain 2D1 was able to accumulate a maximum of 12.55% of putative PHB against the 4.85% produced by the species 1B in the same culture conditions with optimal parameters implemented. Moreover, we investigated the fermentation capability at different argan residue concentrations, and both strains were able to ferment a maximum of 3 g of pretreated argan waste. At higher waste concentrations (4 to 5%), although both species showed increasing growth, the amount of putative PHB final yield measured was lower (4.57% for 1B and 6.50% for 2D1). Finally, for the culture optimization of the species 2D1, a larger culture volume was investigated. With all optimal conditions implemented in a culture medium of 500 mL (10 times higher than the volume used previously), 2D1 was able to accumulate 21.40% of putative PHB, while 1B produced only 14.02%. UV-visible and FTIR analyses indicated that the 2D1 extract contained PHB, showing the relevant peak at 248 nm in UV spectrophotometry and the characteristic peaks at 1726 and 1270 cm^−1^ in FTIR analysis. However, the presence of discordant peaks revealed that further improvement of sample purification during extraction will be required to avoid the presence of impurities that could interfere in the spectral analysis and for further applications of the extract.

To conclude, this work presented a new isolated species from argan tree soil able to produce PHB, as indicated by the characterization analysis. The new isolated species 2D1 revealed better capabilities in sugar and argan seed waste fermentation, as well as in the accumulation of the bio-based polymer, compared to the previously studied 1B. However, concentrations of argan waste greater than 3% lowered the amount of biobased polymer extraction. More research will be required to understand the bacterial effectiveness in waste fermentation, as well as the solubility qualities of the argan seed waste, in order to make better use of the residue. The results presented in this research conducted by using only the seed waste indicated promising routes for exploitation and valorization of the whole argan fruit waste, a costless available raw material used as a sole source of carbon for bio-based polymer production. To follow up on this work, the molecular identification of the new isolated species 2D1 and a further physical and chemical characterization of the bio-based extract will be necessary to better investigate the bacterial properties and the bio-based polymer potential applications.

## Figures and Tables

**Table 1:** Use of different carbon sources for the extraction of the bio-based polymer and analysis of variance.

Carbon Source *	Residual Biomass * g/L	DCW * g/L	Bio-Based Polymer * g/L	Bio-Based Polymer %
Glucose	17.50	18.45 ± 0.13 ^c^	0.95 ± 0.12 ^c^	5.15
Fructose	19.01	19.35 ± 0.23 ^b^	0.34 ± 0.20 ^b^	1.75
Maltose	17.43	17.82 ± 0.15 ^b^	0.39 ± 0.17 ^b^	2.19
Saccharose	20.37	21.25 ± 0.11 ^c^	0.88 ± 0.13 ^c^	4.14
Sorbitol	14.99	15.30 ± 0.15 ^c^	0.31 ± 0.10 ^c^	2.02
Lactose	13.80	14.00 ± 0.20 ^b^	0.20 ± 0.17 ^b^	1.42
Mannose	14.83	15.06 ± 0.19 ^b^	0.23 ± 0.17 ^b^	1.53
Xylose	19.22	20.05 ± 0.10 ^c^	0.83 ± 0.08 ^d^	4.14

* All results in g/L correspond to the amount extracted from 1 g of sugar in 50 mL culture. Data represent mean ± SD from three replicates; in each column, ^b,c,d^ letters indicate significant differences at the *p* < 0.05 level (one-way ANOVA test) using OriginPro 9.0.

**Table 2:** Use of argan seed waste for the measurements of DCW, bio-based polymer, and residual biomass for the strains 2D1 and 1B at 37 °C and 48 h incubation.

Bacterial Strain *	Residual Biomass * g/L	DCW * g/L	Bio-Based Polymer * g/L	Bio-Based Polymer %
2D1	18.57	20.45 ± 0.29 ^a^	1.88 ± 0.35 ^a^	9.19
1B	18.08	18.65 ± 0.11 ^c^	0.57 ± 0.20 ^b^	3.06

* All results in g/L correspond to the amount extracted from 1 g of argan seed pulp in 50 mL culture. Data represent mean ± SD from three replicates; in each column, ^a,b,c^ letters indicate significant differences at the *p* < 0.05 level (one-way ANOVA test) using OriginPro 9.0.

**Table 3:** Gradual increase in DCW and putative PHB production of the species 2D1 in MSM with the selected optimal growth parameters.

Growth Conditions	Selected Optimal Conditions	DCW g/L	Bio-Based Polymer g/L	Bio-Based Polymer %
Temperature *	36°–38 °C	ND	ND	ND
Incubation time *	48 h	21.21 ± 0.21 ^b^	1.92± 0.19 ^b^	9.03
pH *	7.5–8.5	22.67 ± 0.17 ^c^	2.04 ± 0.13 ^c^	8.99
NaCl *	10%	23.75 ± 0.19 ^b^	2.86 ± 0.13 ^c^	12.04
N sources * (0.25%)	Ammonium nitrate	23.58 ± 0.30 ^a^	2.96 ± 0.25 ^b^	12.55
Argan seed waste	3%	23.25 ± 0.25 ^b^	3.26 ± 0.20 ^b^	14.02
MSM volume **	500 mL	27.61 ± 0.09 ^d^	5.91 ± 0.16 ^c^	21.40

Abbreviations: ND, not determined—to select the optimal temperature, the OD was measured. * All results in g/L correspond to the amount extracted from 1 g of argan seed pulp (1%) in 50 mL culture. ** Results in g/L correspond to the amount extracted from 3 g of argan seed pulp (3%) in 500 mL culture. Data represent mean ± SD from three replicates; in each column, ^a,b,c,d^ letters indicate significant differences at the *p* < 0.05 level (one-way ANOVA test) using OriginPro 9.0.

## Data Availability

The data presented in this study are available on request from the corresponding author. The sequence of the 1B species is deposited in GenBank of NCBI with accession number ON584353.
